# Lymphoma Caused by Intestinal Microbiota

**DOI:** 10.3390/ijerph110909038

**Published:** 2014-09-01

**Authors:** Mitsuko L. Yamamoto, Robert H. Schiestl

**Affiliations:** Department of Pathology, Environmental Health and Radiation Oncology, University of California, Los Angeles, Schools of Medicine and Public Health, 10833 Le Conte Ave, Los Angeles, CA 90095, USA; E-Mail: m.l.yamamoto@gmail.com

**Keywords:** intestinal microbiota, inflammation, cancer, lymphoma, prevention

## Abstract

The intestinal microbiota and gut immune system must constantly communicate to maintain a balance between tolerance and activation: on the one hand, our immune system should protect us from pathogenic microbes and on the other hand, most of the millions of microbes in and on our body are innocuous symbionts and some can even be beneficial. Since there is such a close interaction between the immune system and the intestinal microbiota, it is not surprising that some lymphomas such as mucosal-associated lymphoid tissue (MALT) lymphoma have been shown to be caused by the presence of certain bacteria. Animal models played an important role in establishing causation and mechanism of bacteria-induced MALT lymphoma. In this review we discuss different ways that animal models have been applied to establish a link between the gut microbiota and lymphoma and how animal models have helped to elucidate mechanisms of microbiota-induced lymphoma. While there are not a plethora of studies demonstrating a connection between microbiota and lymphoma development, we believe that animal models are a system which can be exploited in the future to enhance our understanding of causation and improve prognosis and treatment of lymphoma.

## 1. Introduction

Lymphocytes play a key role in responding to microbial colonization by initiating an immune response leading to tolerance or activation. The majority of immunologically active cells belong to the mucosal-associated immune system and are constantly receiving signals from dendritic cells or other APCs which are sampling the intestines. Dysregulation can lead to inflammation-related diseases such as colitis and cancer, as reviewed in this issue. Tissues closely associated with bacterial exposure have been most easily identified as being affected by microbes such as colon cancer and gastric cancers [1] and in current issue) however intestinal health can alter extra-gastrointestinal tissues, having a systemic effect [2,3]. Animal models have played an essential role in understanding the importance of the gut microbiome in immune development and composition [4]. Animal models have also played a key role in solidifying the relationship between the microbiome and health and disease [5]. Techniques to manipulate animal gut composition have been studied and refined for over 50 years and continue to play an important role in clarifying this symbiotic and sometimes pathogenic relationship [6].

## 2. Microbiota and Lymphoma in Animal Models

There are two major ways that animal models have an advantage in studying the relationship between gut microbes and cancer. First, the mouse gut microbiome can be altered to be germ free, contain specific species of bacteria (gnotobiotic), or to have what is commonly called conventional microbiota, which is considered “normal” and generally unmonitored in genetically similar animals. Changing the microbiome allows us to study cause and effect relationship between the bacteria and body. Germfree animals have demonstrated the role of microbiota in inflammation, metabolism, and obesity [5,7,8]. Gnotobiotic models have helped to determine both causative species and mechanisms of colorectal cancer [9,10]. Second, animal models have been used to determine how genes may affect or be affected by different bacteria. These models can help us determine genetic susceptibility or resistance to different diseases depending on microbial exposure. For example polymorphisms in Dectin1 can influence susceptibility to colitis [11]. Alternatively, genetic models can help us determine which genes or pathways may be important in disease development or protection [12]. For example, Rag2-/- mice can develop *H. hepaticus*-induced cancer, however immune competent mice are protected due to a regulatory response leading to decreased inflammation [13]. Combining both a defined gut microbiota and genetic models can also give us important insights into mechanisms of gut-microbe interactions.

### 2.1. Historical Data Indicating That Gut Microbes May Affect Mouse Phenotypes Such as Cancer and Lifespan 

While inbred mouse strains have helped to decrease variability among and within experiments, over time even these carefully maintained strains may acquire differences. Changes in phenotypes of research animals have been noted as early as 1966 in various fields from radiation to toxicology [14,15,16,17,18]. Internal factors such as genetic drift or spontaneous mutations can play a role in varying phenotypes of research animals [19,20]. Many environmental factors have also been postulated to contribute to changes in rodents including housing conditions, diet, and sterility [15,17,21].

Significant changes in lifespan or tumor incidence may have important consequences on experimental results. For example, over an 11 year period, percent survival of male F344 rats at 106 weeks decreased from 85.3% in experiments starting in 1971 to 62.5% in experiments starting in 1980–1981 [16]. In addition, leukemia incidence increased from 9.4% to 20.1% in male rats in experiments starting in 1972–1973 and 1980–1981, respectively [16]. Experiments using 3 Gy X-rays to induce myeloid leukemia show that 32.3% of mice developed the disease in 1956 while only 12.8% developed myeloid leukemia in 1964 [17]. While different environmental factors have been attributed to these changes, it is well known that animal husbandry protocols have also become more stringent, affecting animal microbial composition and health [22]. More recently, our lab has shown that in different vivariums, with different specific pathogen free (SPF) conditions, isogenic mice have altered lifespans and lymphoma latency periods [18,23]. This correlates to distinct microbiome profiles as determined by 16S rRNA lengths. Therefore it is likely that the microbiome has at least a partial influence on animal health, including carcinogenesis.

### 2.2. Animal Models of Mucosal-Associated Lymphoid Tissue (MALT) Lymphoma

Mucosal-associated lymphoid tissue (MALT) lymphomas are thought to originate in the marginal zone and are strongly associated with the presence of *Helicobacter* [24,25]. Approximately 90% of MALT lymphomas are associated with *Helicobacter* infection [26]. Elimination of *Helicobacter* leads to complete remission in approximately 80% of all cases [27]. While the association of *H. pylori* and MALT lymphoma was discovered in humans, the causative effect of *Helicobacter* in MALT lymphoma development, according to the Koch’s Postulate, was demonstrated in animal models. A model of bacteria-induced MALT was first shown in mice by infection with *H. felis*, a close relative to *H. pylori.* Twenty two weeks post-infection, 25% of infected mice had lymphoepithelial lesions while none of the non-infected animals did [28]. An *H. pylori* infection was first established in gerbils and showed an increase in gastritis and intestinal metaplasia [29]. Since then, *H. pylori* infections have been established in mouse models and have been used to examine mechanism by assessing transcription profiling [30] and disease progression and regression [31].

*H. helmanii*, found in both human and mice, also leads to MALT lymphoma which is preceded by inflammation and high endothelial venule-like vesicles, which are associated with lymphocyte recruitment and present in other chronic inflammatory conditions such as rheumatoid arthritis, and colitis [32]. The animal models of *H. helmanii*-induced lymphoma, however, seem to have varying results and may also involve host and bacterial factors [33]. The use of better defined bacteria, however, may improve consistency and development of MALT lymphoma for future studies [34].

Other bacteria such as *Campylobacter jejuni*, *Borrelia bergdorferi*, and *Chlamidia psitacci* may also play a role in lymphoma development, however these associations have only been shown in humans thus far [35]. *Streptococcus bovis* has been associated with hematopoietic malignancy in humans [36]. Therefore, animal models may provide valuable insight into microbe-associated lymphoma etiology, progression, and treatment.

### 2.3. Animal Models of Lymphoma and Effects of the Microbiome

Animal models of cancer can also be useful in demonstrating a link between the microbiome and carcinogenesis. Cancer is a disease that is generally thought to occur in a multi-step process beginning with initiation, promotion, and finally progression. As the disease progresses, cells acquire “hallmarks of cancer” which include sustained proliferation, resistance to cell death, and metastasis [37]. Using animal cancer models such as p53-deficient mice, allows researchers to bypass some steps required for overt cancer saving time and animal numbers. ApcMin/+ mice, which spontaneously develop intestinal polyps, have been used to demonstrate that infection with *Citrobacter rodentium* or enterotoxin-producing *Bacterioides fragilis* can promote colon cancer [38,39]. A chemically induced model of liver cancer also showed that *Helicobacter hepaticus* infection promotes liver tumorigenesis [40].

Our lab has shown that mice deficient in the Ataxia telangiectasia mutated gene (Atm-/- mice), which display genetic instability and spontaneously develop a high incidence of thymic lymphoma [41,42], are sensitive to changes in microbial content [23]. We found that as *Atm*-/- mice moved to more sterile conditions, they began to live longer and have a decreased lymphoma penetrance [18,23]. Conversely, when they were moved to standard SPF conditions, their lifespan and lymphoma latency decreased. To test the effects of the gut microbiota more directly, we rederived mice into a restricted microbiota facility [43] and “conventionalized” mice by inoculating them with fecal samples from conventional SPF mice. Again, the “conventionalized” mice had shorter lifespans than the mice with a restricted flora [23]. These results indicated that microbes in the restricted, sterile facility had a protective effect in *Atm*-/- mice and/or the conventional microbiota had a more pathogenic effect. One microbe that was highly enriched in the restricted microbiome was *Lactobacillus johnsonii*. Inoculation with *L. johnsonii* in *Atm*-/- mice decreased measures of DNA damage, oxidative stress, and inflammation [23]. These results indicate that the gut microbiota can impact lymphomagenesis in *Atm*-/- mice. Other lymphoma or cancer models may also contribute to the growing body of evidence linking the microbiome to carcinogenesis.

## 3. Mechanism of Microbiota-Induced Lymphomagenesis as Evidenced in Animal Models

While there is not a plethora of animal models linking the microbiome to lymphoma development, there is a large amount of data indicating plausible mechanisms of microbiota-induced lymphomagenesis in animal models. Since the intestinal microbiota has been shown to influence the immune system directly and indirectly ([44,45] and in the current issue), there are several ways that the intestinal microbiota may affect lymphomagenesis in mice. Many of these mechanisms have been identified and shown in animal models.

### 3.1. Microbiota Can Directly Initiate Lymphomagenesis

Species of gut bacteria may directly cause the promotion or neutralization of mutagens and oxidative stress [46,47,48,49,50,51,52,53,54] leading to DNA damage and subsequent cancer or protection [55] From here on the reference No need to be shifted for one number see attached original. Faecal water samples from mice treated with pre- and probiotics showed different degrees of genotoxicity which correlated with tumorigenesis [55]. Bacteria can also directly interact with immune cells causing oxidative bursts [56] or necrosis [56], and with epithelial cells causing increased production of reactive oxygen species and inhibition of NF-κB [57]. Oxidative stress can then lead to DNA damage and carcinogenesis [58,59,60]. *H. pylori* and *C. jejuni* have both been shown to increase oxidative stress [53,54]. Finally, bacteria can act as an antigen and stimulate chronic proliferation of immune cells. *H. pylori* is thought to cause lymphoma because of constant stimulation of antigen presentation leading to B cell expansion [31,61]. In humans, this is evident in the overrepresentation of certain V genes [35,62]. While the lymphocytes and microbes are generally separated by the epithelial barrier, bacteria, antigens, or metabolites cross the mucosal barrier through dendritic cells or M cells which are constantly sampling the lumen [63]. In addition to causing damage, bacteria can also help to neutralize mutagens and oxidative stress ([64], reviewed in [65]). The mutagens MNNG and DMH have been shown to be neutralized in rat colons by lactic acid bacteria.

### 3.2. Microbiota Can Alter Immune Parameters to Affect Lymphomagenesis

Species, or populations of gut bacteria may cause a change in immune response or immune parameters and affect lymphocytes. Intestinal immune cells are constantly sampling luminal content and deciding whether to elicit or suppress an immune response [44]. Animal studies have shown that both single species of bacteria as well as different bacterial compositions can have large impacts on immune parameters. For example, studies using germ-free mice established that intestinal microbiota are essential for normal immune system development [66,67,68]. Since lymphoma itself is a shift in immune cell types, it is not surprising that microbes may influence lymphomagenesis.

Several animal studies have shown that either a mixture of bacteria or single species may significantly affect immune cell population and activity [43,69,70,71,72,73]. For example, inoculation with segmented filamentous bacteria caused a change in T cell activity eliciting a range of responses including increases in IL-10, IL-17, and IFN-γ [72]. In addition, inoculation of *Sphingomonas yanoikuyae* caused a systemic change in immune cell populations [43]. *Bacterioides fragilis* can induce a Th17 response in mice which was then shown to be required for tumorigenesis [39]. Bacteria can also directly alter inflammation-related pathways. Inoculation with common human commensal bacterium *B. thetaiotaomicron*, *B. longum*, or both resulted in an increase in TNF-α- and IFN-γ-associated pathways [74]. These studies indicate that gut microbes can affect the immune system which may impact lymphoma development.

Alternatively, distinct compositions of intestinal microbiota can differentially alter immune parameters [43,69,70] which may protect mice against cancerous cells. Mice with a restricted microbiota have increased cytotoxic T cells which leads to decreased levels of marginal zone B cells [69], invariant NKT (*i*NKT) cells [70], and plasmacytic dendritic cells compared to mice with conventional flora [43]. Moreover, Wei *et al.* suggest that the activity of adoptively transferred cytotoxic CD8+ T cells can be increased if recipient mice are inoculated with donor microbial antigens [69]. It has also been shown that germ-free colorectal cancer rat models mount different responses to cancer induction compared to conventional mice including increased B cells, NK cells and cytotoxic T lymphocytes [75].

Conversely, some bacteria and bacterial products may have a beneficial effect. For example, lactic acid bacteria and specific recognition of Lactobacilli may protect against carcinogenesis [76,77]. *Lactobacillus johnsonii* in rat intestines has been shown to have a positive effect on oxidative stress and inflammation and prolongs the development of diabetes [78,79]. Lactic acid bacteria can also modify the immune system to prevent cancer in mouse tumor models ([80,81,82] (reviewed in [65,83]). In addition, butyrate, a short-chain fatty acid produced by bacterial fermentation of fiber on Treg cell specification and expansion [84,85].

Whether microbes influence immune cells directly, indirectly, or a combination of both, increased lymphocyte proliferation can lead to a higher chance of aberrant DNA replication [86,87], particularly in some B lymphocytes which are innately vulnerable to genetic instability [88,89] and activation [90]. Oxidative stress caused by intestinal microbiota either directly [91] or indirectly through the immune system [92], can also affect carcinogenesis. Therefore, the microbiota can affect several pathways associated with lymphomagenesis [93,94].

## 4. Conclusions

While there is evidence that the microbiome affects lymphomagenesis, particularly MALT lymphomas, there is a wide gap of knowledge to which animal models could provide valuable answers. Namely, which bacteria or bacterial products can cause, protect against, or increase risk of lymphoma development? With the exception of breast cancer, liver cancer, and lymphoma, systemic effects of intestinal bacteria on cancer have not been studied. Lymphomas are of particular interest because they circulate through the gastro-intestinal system as well as the rest of the body. The fact that more lymphomas are becoming associated with bacterial infections [95,96,97] and that antibiotic therapy can be effective [95,97] underscores the need for more studies involving microbes and lymphoma.

There is overwhelming evidence that some intestinal bacteria are health beneficial like the Lactobacilli whereas some others are health detrimental like some of the *Helicobacteraceae*. It will be very important to determine the roles as health beneficial and detrimental of most intestinal bacteria and whether there are synergisms or antagonisms between them. Then one can design certain probiotics containing the health beneficial and certain antibiotics against the health detrimental bacteria.
